# In-Silico Lead Druggable Compounds Identification against SARS COVID-19 Main Protease Target from In-House, Chembridge and Zinc Databases by Structure-Based Virtual Screening, Molecular Docking and Molecular Dynamics Simulations

**DOI:** 10.3390/bioengineering10010100

**Published:** 2023-01-11

**Authors:** Mehreen Ghufran, Mehran Ullah, Haider Ali Khan, Sabreen Ghufran, Muhammad Ayaz, Muhammad Siddiq, Syed Qamar Abbas, Syed Shams ul Hassan, Simona Bungau

**Affiliations:** 1 Department of Pathology, Medical Teaching Institution Bacha Khan Medical College (BKMC) Mardan, Mardan 23200, Pakistan; 2 District Medical Officer, Sehat Sahulat Program (SSP), KPK, Mardan 23200, Pakistan; 3 Mardan Medical Complex (MMC) Mardan, Medical Teaching Institution Bacha Khan Medical College (BKMC), Mardan 23200, Pakistan; 4 Department of Biochemistry, Abdul Wali Khan University Mardan, Mardan 23200, Pakistan; 5 Department of Pharmacy, University of Malakand, Chakdara 18000, Pakistan; 6 Department of Pharmacy, Abdul Wali Khan University Mardan, Mardan 23200, Pakistan; 7 Department of Pharmacy, Sarhad University of Science and technology, Peshawar 25000, Pakistan; 8 Shanghai Key Laboratory for Molecular Engineering of Chiral Drugs, School of Pharmacy, Shanghai Jiao Tong University, Shanghai 200240, China; 9 Department of Natural Product Chemistry, School of Pharmacy, Shanghai Jiao Tong University, Shanghai 200240, China; 10 Department of Pharmacy, Faculty of Medicine and Pharmacy, University of Oradea, 410028 Oradea, Romania

**Keywords:** main protease (M^pro^), structure-based virtual screening, ZINC, in-house, ChemBridge database, molecular dynamics simulation

## Abstract

Pharmacological strategies to lower the viral load among patients suffering from severe diseases were researched in great detail during the SARS-CoV-2 outbreak. The viral protease M^pro^ (3CLpro) is necessary for viral replication and is among the main therapeutic targets proposed, thus far. To stop the pandemic from spreading, researchers are working to find more effective M^pro^ inhibitors against SARS-CoV-2. The 33.8 kDa M^pro^ protease of SARS-CoV-2, being a nonhuman homologue, has the possibility of being utilized as a therapeutic target against coronaviruses. To develop drug-like compounds capable of preventing the replication of SARS-main CoV-2’s protease (M^pro^), a computer-aided drug design (CADD) approach is extremely viable. Using MOE, structure-based virtual screening (SBVS) of in-house and commercial databases was carried out using SARS-CoV-2 proteins. The most promising hits obtained during virtual screening (VS) were put through molecular docking with the help of MOE. The virtual screening yielded 3/5 hits (in-house database) and 56/66 hits (commercial databases). Finally, 3/5 hits (in-house database), 3/5 hits (ZINC database), and 2/7 hits (ChemBridge database) were chosen as potent lead compounds using various scaffolds due to their considerable binding affinity with M^pro^ protein. The outcomes of SBVS were then validated using an analysis based on molecular dynamics simulation (MDS). The complexes’ stability was tested using MDS and post-MDS. The most promising candidates were found to exhibit a high capacity for fitting into the protein-binding pocket and interacting with the catalytic dyad. At least one of the scaffolds selected will possibly prove useful for future research. However, further scientific confirmation in the form of preclinical and clinical research is required before implementation.

## 1. Introduction

Since December 2019, after the first outbreak of Corona virus infection reported from Wuhan, China, the disease has devastated life throughout the world and the search for affective therapeutics is underway [1]. The virus is known as SARS-CoV-2 because its RNA genome shares 82 percent of its sequence with the SARS Corona virus [2]. These viruses are related to the betacoronavirus clade B [3,4]. Although the outbreak was initially thought to have originated in Wuhan’s Huanan seafood and cattle market, effective human-to-human transmission has caused the number of patients to rise dramatically. As of April 9th, there were more than 1,500,000 diseased individuals with a 5.9% mortality rate. The recent emergence of the coronavirus-2 causing severe acute respiratory illness (SARS-CoV-2) has led to the global pandemic of coronavirus disease 2019 (COVID-19). By April 2021, there had been more than 140 million infections reported, causing more than 3 million fatalities globally. Antiviral medications will likely be essential to manage the anticipated future outbreaks of coronaviruses, despite the promising COVID-19 immunization campaigns. The emergence of SARS-CoV-2 variants for which vaccinations are ineffective suggests that antiviral medications will eventually be needed to enhance immunizations [5]. Similar to the common cold virus, SARS-CoV-2 is expected to continue spreading and provide a significant threat to our society. In this condition, antiviral medications are required to treat infected patients as well as be delivered prophylactically to protect high-risk groups. Since therapeutic medicines that suppress coronavirus replication have the potential to enhance the lives of millions of people throughout the world, their discovery must be prioritized despite the lengthy drug development process. After SARS-CoV-1 (found in 2002) and MERS-CoV (Middle East respiratory illness, 2012), SARS-CoV-2 is the deadliest of the zoonotic coronaviruses that have infected humans [6]. Similar to other coronaviruses, SARS-CoV-2 affects the respiratory system and causes severe pneumonia, which necessitates ventilatory assistance and intensive care, especially in the elderly and immunocompromised patients [3]. Vaccine development has advanced significantly, but supply and timing are currently limiting factors for its effective implementation. Several vaccinations have been developed and licensed for mass immunization [7]. The cost of storing some vaccines at cryogenic temperatures, meanwhile, may be prohibitive in underdeveloped nations. Additionally, a number of changes to the SARS-CoV-2 genome may impact how well vaccines work to fight the virus [8,9]. These results highlight how critical it is to simultaneously develop therapeutic options for SARS-CoV-2 treatment.

SARS-CoV-2 belongs to the beta group of coronaviruses, an RNA virus with only one strand. It has structural proteins, such as spike-like protein S and lipid membranes, as well as M protein (membrane), N protein (nucleo-capsid), and envelope (E) protein that give it an envelope appearance. The S spike protein binds to the angiotensin-converting enzyme 2 (ACE2) receptor on mammalian lung cells, allowing the virus’s RNA genetic material to be released into the host cells [10]. Four nonstructural proteins are found in the virus: papain-like (PLpro) and CoV main proteases [M^pro^; also known as 3CLpro] [11], RNA polymerase, and helicase [12]. The virus’s transcription and replication are aided by both proteases (PLpro and 3CLpro). The replicase genes encode two polyproteins important for effective viral replication and transcription [13]. A significant proteolytic process liberates the functional polypeptides from these two polyproteins (pp1a and pp1ab). Proteolysis is mostly performed by a papain-like protease (PLpro), which cuts proteins in three places, and a 33.8 kDa main protease (Mpro), also called a 3C-like protease, which cuts proteins in 11 sites, making nonstructural proteins in the process (NSPs). As no host protease recognizes the M^pro^ recognition sequence, to develop drugs against SARS-CoV-2 infections, this enzyme is a prime target for the development of inhibitors. The Mpro and PLpro enzymes, which digest viral polyproteins produced by the host cell translational machinery, build a functionally active viral replication complex and package it into host cells during viral replication [14]. Three domains make up the 3CLpro monomer, with the active site (Cys 145 and His 41) located between domains I and II. A larger pocket is found in the gap between the third domain and the protein structure because of the long loop connecting it to the rest [15]. Additionally, 3CLpro is the main protease of the virus, and it helps in the replication of the virus, making it a valuable antiviral treatment target. M^pro^ is a crucial target for antiviral medication because the human genome lacks a homologue of it. Although there is no homologue of M^pro^ in the human genome, it is known as 3CLpro (3-chymotrypsin-like protease) and aids in viral replication, making it a significant target for antiviral treatments. Protease inhibitors efficiently stop coronavirus replication and proliferation by obstructing the post-translational processing of essential viral polypeptides [16]. Pfizer’s PF-07321332 is an oral antiviral compound that is designed to stop SARS-CoV-2 Mpro from modifying the active site Cys145 with its nitrile warhead. It is thought to be a good antiviral candidate and is currently being tested (NCT04756531, NCT04909853, NCT05011513, and ClinicalTrials.gov (accessed on 23 March 2021)). The oral antiviral PAXLOVIDTM, which is a combination of PF-07321332 and the HIV drug ritonavir, which slows down the breakdown of PF-07321332, was found to reduce the risk of hospitalization or death by 89% compared to a placebo in nonhospitalized high-risk adults with COVID-19. In December 2021, the FDA gave Pfizer’s Paxlovid an emergency use authorization to treat mild-to-moderate COVID-19 in adults and children older than 12 who are at least 12 years old (www.fda.gov) [17]. Crystallization of the main protease of SARS-CoV-2 (PDB ID: 6LU7) has been accomplished by Liu et al., which provides an opportunity to combat the disease by identifying it as a potential therapeutic target. When opposed to methods based on trial and error that involve experimental research, the use of the in silico method for the screening of prospective therapeutic compounds has been demonstrated to be both time and cost efficient. The in silico method of molecular docking has the capacity to screen and find potentially useful therapeutic compounds from large and huge compound databases. At the moment, several molecular docking studies are being conducted against SARS-CoV-2 receptors with drug-like compounds (ChemBridge, ZINC, and in-house databases). Furthermore, the majority of these docking investigations used quantitative structure-activity relationships (QSARs) modeling, similarity searches, and structure-based drug design (SBDD) [18].

Drug repurposing is one method for speeding up the normal drug development and drug discovery process. This will help to clarify the effectiveness of novel therapeutic uses for substances whose efficacy and safety have already been established. The importance of potential therapeutic compounds that function as efficient antivirals in controlling the pandemic is highlighted by the increasing and faster spread of SARS-CoV-2 as well as the appearance of novel variants. In the formation of anticoronavirus therapeutics, M^pro’^s catalytic activity may be hindered. In light of this background, the current research looked for a few phytocomponents that could suppress the M^pro^ protein. Phytochemicals are used as active drugs in drug development. In the fight against viral diseases, phytocompounds derived from a variety of medicinal plants may enhance immune function and combat pathogens. Phytochemicals and their derivatives have been the focus of numerous studies due to their antiviral activities and mechanisms of action, which have been demonstrated to be crucial in the treatment of viral diseases [19]. Over the past decade, computer-aided drug discovery (CADD) approaches have emerged as a crucial component of the drug development process, having been used to identify protein inhibitors and investigate interactions between proteins and drugs and proteins themselves [20]. Despite the time and money required to develop a candidate drug into an approved drug, computational methods, such as virtual screening, docking, molecular dynamics (MD) simulations [21], and binding free energy evaluation, can be used to identify promising drug candidates from compound libraries. The purpose of this research was to find potential anti-SARS-CoV-2 treatments by utilizing a multipronged strategy that included both molecular docking and virtual screening techniques. The information that is obtained from the screening will be helpful in the investigation of new inhibitors of the SARS-CoV-2 M^pro^ target that have the potential to be both effective and selective.

## 2. Materials and Methods

### 2.1. Protein Preparation

A worldwide database called the Protein Data Bank (PDB) was used to find the three-dimensional structure of main protease (M^pro^) with co crystallized ligand structure, which causes severe acute respiratory syndrome (PDB Id: 6LU7) (accessed on 23 March 2021) (https://www.rcsb.org). The main protease is composed of two chains, such as A and C. Chain A is the protease, and chain C is the N-[(5methylisoxazol-3yl) carbonyl] alanyl-L-valyl-N-1-(1R,2Z) (1R,2Z)-4-(benzyloxy) (benzyloxy) [(3R)-2-oxopyprolidin-3-3yl]-4-oxo-1-1-[(3R)-2-oxopyprolidin-3-3yl] but-2-enyl methyl)-L-lucinamide (N3 inhibitor). A peptide inhibitor (N3 inhibitor) was in a complex with the protein. Water molecules, inhibitors, and other heteroatoms were removed from the protein structure. All the atoms’ Amber14:EHT (Amber ff14SB and ETH combined) forcefields were used to refine the protein structure. The missing hydrogens were added to the amino acids. The forcefield parameters, missing atom types, bond stretch parameters, missing angles, and missing van der Waals parameters were added to all atoms. Restrained electrostatic potential atomic partial charges (RESP) and AM1-BCC [22] charges were used for the protein-ligand complex, the RESP, and AM1-BCC charges were used for leap protein preparation and for preparation of inhibitors, respectively. The 3D protonation was used to incorporate hydrogen atoms into the protein structure, followed by minimization of energy with the MOE (molecular operating environment) program’s default parameters [23].

### 2.2. Preparation of Databases

Small molecules used in virtual screening can be found in commercial databases, such as ZINC and ChemBridge. ZINC had millions of compounds, compared to the ChemBridge database that contain 168423 ligands/compounds in ChemBridge [24]. A Tanimoto cut-off level of 60% was used to screen the ZINC database, which resulted in the production of a library containing 11,193 drug-like molecules [25]. An in-house database containing compounds isolated or synthesized by our collaborators, with a focus on natural products and structural analogues. The in-house database contains over 1600 compounds, representing a wide range of structural diversity across a wide variety of core scaffolds and substitution patterns. Three-dimensional protonation (MMFF94x force field) and energy minimization (constrained minimization of 0.01 Kcal/Å^2^ was performed to optimize the ligand structures) using MOE were performed on all of the compounds in the in-house database. Anti-COVID-19 lead compounds can be found using structure-based virtual screening, which scans both in-house and commercial databases.

### 2.3. Structure-Based Virtual Screening

The drug target (receptor) and ligands’ 3D structures in the database are necessary for the structure-based virtual screening (SBVS) methods. In order to find new potential inhibitors, we employed molecular docking approach to assess the binding modes of drug target proteins and the ligands, called the structure-based drug design (SBDD). This helps to predict the improved and healthy interactions that will take place between the target receptor and the drug. Using the MOE, the SBVS was used to screen ZINC, in-house, and ChemBridge databases. Additionally, using both revised-Lipinski’s rule of 5 as well as Lipinski’s rule of 5, the number of screened results was significantly reduced. New lead hits discovered through screening a compound database of thousands of compounds are illustrated by the property of “drug-likeness” [26].

### 2.4. Molecular Docking

The MOE docking software was utilized to conduct the molecular docking studies [27]. The retrieved compounds were docked with M^pro^ to further evaluate these drug-like compounds. The 3D protonation of the target receptor was followed by energy minimization using the MOE software 2019’s default parameters to achieve the best possible outcome. To improve the result, all of the compounds were docked into the M^pro’^ binding pocket. MOE was used to dock the retrieved hits against the M^pro^ drug target. Ten conformations were generated for each hit, with the top-ranked conformations of each inhibitor being used for advanced research. The docking analysis was scrutinized more closely, with docking scores and protein/hit interactions playing a larger role. In addition, the results of molecular docking were validated using MDS.

### 2.5. Molecular Dynamics Simulation (MDS)

A molecular dynamic (MD) simulation was carried out to investigate the dynamic behavior of proteins upon inhibitor binding at the atomic level. The docked conformations of the selected hits within the active pocket of M^pro^ were subjected to MD simulations. A detailed MDS analysis was carried out using the Amber14 package and the ff14SB force field [28]. To evaluate the stability of the previously retrieved compounds at the active sites of M^pro^ and M^pro^/N3 complex, MDS was used. Tleap, a preparatory program, was used to build and solve the complexes. The solvated octahedral box was used in this experiment. After solvating each system in an octahedral box using the TIP3P water model with 15 Å, the systems were neutralized by adding counterions (either Na+ or Cl−). Each neutralized system’s energy was reduced as much as possible through two steps of energy minimization in order to achieve the goal of relaxing all of the systems. These steps were steepest descent minimization and conjugate gradient minimization. At 50 ps, the minimized complexes were heated to 300 K. Then, using a two-step process, each system was brought into equilibrium at a constant 1 atom and 300 K. First, we used a weak restraint to equilibrate the density at 50 ps. Second, we equilibrated the system without any constraints for 1 ns. After that, the production step was run for 150 ns. To keep the temperature stable, the Langevin thermostat was activated [29] and Berendsen barostat was used to monitor the system pressure. For the calculation of long-range electrostatic interaction, we used the AMBER18 Particle Mesh Ewald (PME) algorithm. A cut-off distance of 10 Å was used for long-range electrostatic interactions and van der Waals interactions. The covalent bonds were refined using the AMBER18 SHAKE algorithm [30]. The GPU version (PMEMD.cuda) [31] of AMBER18 was used to run MD simulations on four complexes with M^pro^/N3 complexes. The AMBER18 CPPRTAJ module was used to analyze the MD trajectories. The interface analysis and graphical representation were carried out using MOE2019 software, PyMol v1.7, and Origin Pro Lab v2018.

### 2.6. Assessment of Binding Free Energy

For the calculation of binding free energy (BFE), trajectories generated by molecular dynamics simulations using the MMPBSA.py script were used [32]. Numerous studies have employed this method to evaluate the binding free energies of P-P (protein-protein), protein-ligand, and nucleic acid-protein complexes. The total binding free energy (Gbind) was calculated with the help of the following Equation:

ΔGbind=ΔGcomplex−[ΔGreceptor+ΔGligand]

For each of the energy terms, for example, polar (Gpol), van der Waal forces (GvdW), electrostatic energy (Gele), no-polar interactions (Gnpol),Gbond showed the angle of bond and their dihedral energy, TS represents the absolute temperature (T) and entropy (S), the equation below was used to better understand how they contribute to the total energy (G).

G = Gele + Gbond + GvdW + Gnpol + Gpol − TS

The molecular mechanics generalized born surface area (MM-GBSA) method was used to calculate the binding free energies of the retrieved compounds/complexes and M^pro^/N3. Since the MM-GBSA is a BFE index, the lower the value, the stronger the bond. The binding free energy of the retrieved and reference complexes was calculated in Amber 18 using the python script MMGBSA.py. The decrease in potential energy over 150 ns revealed that the system is stable in the case of complexes. The various conformations obtained over a 150 ns simulation period are examined. The MMGBSA.py script was used to calculate the BFE between M^pro^ and the retrieved hits, as well as the reference drug (N3, peptide inhibitor) [33]. In this study, the MMGBSA scripts from AMBER and AMBER Tools were used to carry out various steps required to assess the BFE of the protein-ligand complex via MMGBSA methods. By taking 15,000 snapshots over a 150-ns trajectory, the BFE was calculated.

## 3. Results and Discussion

Despite the encouraging vaccination programs against COVID-19, the use of antivirals is likely going to be necessary in order to contain the unpredictable outbreaks of coronaviruses that will occur in the future. Vaccines have already been developed, and there are SARS-CoV-2 variants that are resistant to them, which is clear evidence that antivirals will eventually need to be used in addition to vaccines [5]. SARS-CoV-2 must, therefore, be controlled using an antiviral drug that is both affordable and effective. To discover new drugs, it is helpful to see if existing drugs or drugs with similar properties are effective in the treatment of viral infections. The traditional methods of drug discovery take a long time and are inefficient [34]. According to an in silico study, the N3 inhibitor blocks the active catalytic site of HCov-NL-63, preventing its biological function [35]. The current project aimed at performing structure-based virtual database screening, molecular docking, and drug-likeness evaluations of potential compounds. With the help of this methodology, potent drug candidates were found to bind closely to the M^pro^ of the SARS-CoV-2 catalytic site and limit its proteolytic activity. Through simulation approaches, potential anti-SARS-CoV-2 M^pro^ can be identified by using the structure of the COVID-19 virus M^pro^ in complex with N3. SARS-CoV-2 M^pro^ candidate inhibitors were virtually tested using three databases (ChemBridge, ZINC, and in-house).

### 3.1. Structure-Based Virtual Screening

One of the most useful and effective in silico techniques for the drug design process is the structure-based virtual screening (SBVS) method. SBVS makes an effort to anticipate the interaction mode that will result in the formation of a stable complex between two molecules. It does this by employing scoring functions, which measure the force of noncovalent interactions that occur between a receptor and an inhibitor. Therefore, scoring functions are the primary factors that determine whether or not SBVS software is successful. It is possible to get different results from different software programs, even when utilizing the same input, because these programs all employ different algorithms to perform SBVS, which means that there are many distinct software programs that are used to perform SBVS. In SBVS, the three-dimensional structure of the target protein is already known, and the purpose of the process is to choose ligands from a candidate database in such a way that they will have a greater affinity for the three-dimensional structure of the target. Molecular docking is a computer approach that can be used to perform VS. During this procedure, ligands are moved around in three-dimensional space in an effort to locate a target and ligand combination that maximizes the scoring function. The ligands in the database are ranked according to the highest score they received, and the highest-scoring ones are the ones that can be explored further. For instance, one could look at the mode and kind of interaction that takes place [36]. Through in silico screening, lead inhibitors for the COVID-19 virus M^pro^ can be found by using the structure of the COVID-19 virus M^pro^ in complex with N3. To do this, MOE was used to dock possible binding compounds from the ChemBridge, ZINC, and in-house databases. All the compounds from the different databases (ChemBridge, ZINC, and in-house) were used for virtual screening using MOE software with the M^pro^. Finding hits/compounds that were chemically and structurally comparable required the SBVS of 3D databases, including the in-house, ChemBridge, and ZINC databases. To find prospective, potential, and new inhibitors, VS was performed on the in-house database, which included 1600 ligands, as well as the ZINC and ChemBridge databases. Five hits from the in-house database, 50 hits from ChemBridge, and 16 hits from the ZINC database were all reported by the SBVS. To verify the draggability of the hits, the retrieved results from the commercial database were also subjected to Lipinski’s rule of five, while the results from the in-house database were treated with a modified version of Lipinski’s rule of (5) five. According to Lipinski’s rule of (five) 5, druggable molecules must have the log S-score ≤5, a MW <500 Dalton, an HBA of <10, a log p-score of ≤5, and an HBD of <5. These factors are all indicators of H-bond donors. Molecules which did not fit these requirements; their absorptions would be unsatisfactory [37]. On the other hand, the “modified Lipinski’s rule of five” suggests that molecules having MW >500 logP, HBD greater than 5, and HBA greater than 10 exhibit good absorption. After running the retrieved hits via both Lipinski’s rules [38], it was discovered that, retrieved hits from 49/50 ChemBridge database, 7/16 ZINC database, and 3/5 (in-house database) were following both rules effectively. Subsequently, the 3/5 (in-house) and 56/66 (commercial databases) retrieved hits were further decreased for further analysis using a molecular docking strategy.

### 3.2. Molecular Docking

**Figure 1:**
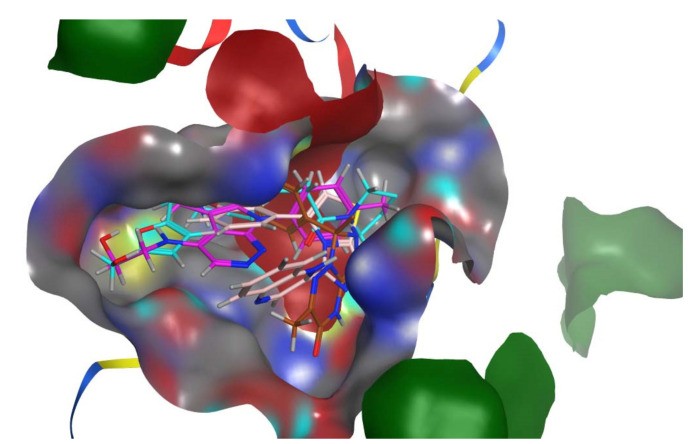
The M^pro^ protein’s molecular surface representation with an overlay of all retrieved active hits in the binding pocket. The ZINC44928678, ZINC08535852, 12-quinoxaline derivative, and ChemBridge63310525 active ligands were represented by purple, dark brown, pink, and cyan colors, respectively.

By docking all the hits from the 3/5 (in-house database) and 56/66 (commercial databases) using the M^pro^ binding pockets via MOE, results in this study were further reduced and refined. In comparison to the standard drug (N3), the docking scores of our refined hits were considerably good (Table 1). For each ligand in the retrieved findings, ten distinct conformations were generated, and all hits having high conformations were sorted and kept in a database file for subsequent analysis. We observed that 3/5 in-house hits, 7/49 ChemBridge database hits, and 5/7 ZINC database hits were best on the basis of docking scores and, thus, were selected for further analysis. Furthermore, the top-ranked conformations of 3/5 hits (in-house), 3/5 hits (ZINC database), and 2/7 hits (ChemBridge database) were well accommodated inside the active site of the M^pro^ drug target and were implicated in several interactions at the active sites of the target protein. From three different databases (ChemBridge, ZINC, and in-house database), docking calculations revealed eight chemically varied molecules having a better binding affinity towards SARS-CoV-2 M^pro^ as compared to N3. Docking studies indicated that the M^pro^ drug target exhibits better docking scores and considerable polar contacts with the hits due to the presence of electronegative capabilities. Table 1 shows the results of molecular docking. The retrieved potential antiviral (anti-COVID19) hits were found to be well-fitted inside the M^pro^ drug target (Figure 1). The compounds reported in Table 1 were ChemBridge, ZINC, and in-house database compounds, and these retrieved compounds were piperidine derivative, tetrahydrothiophene derivative, triazin analog, pyridazine derivative, triazolo pyridine and quinoxaline derivatives, respectively. These compounds have a role in the inhibition of the M^Pro^ target [39].

### 3.3. Analyses of the Binding Interactions of Finally Selected Drug-like Compounds

It is widely recognized that molecular docking provides essential guidelines for the design and discovery of novel drugs. The S-score quantifies the strength of the receptor-ligand interactions. The compounds can be chosen as good drug compounds based on their docking score (S-score). According to the docking study, all finalized hits exhibited favorable contacts with the residues of the M^pro^ target’s binding site when compared to the positive control (Table 2).

**Figure 2:**
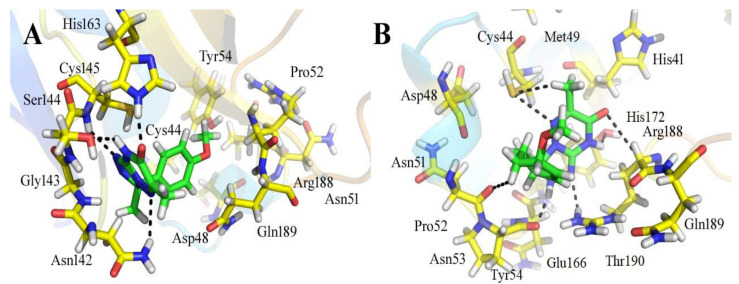
The binding mechanism of the ZINC08535852 ZINC database ligand within the active site of the M^pro^ protein (**A**) before MDS and (**B**) after MDS.

Out of all of the hits identified by using the SBVS against the ZINC, ChemBridge, and in-house databases, ZINC08535852 (ZINC database), with a docking score of −41.3801, was the most active ligand of the ZINC database and demonstrated strong interactions with the active site residues of the M^pro^ of SARS-CoV-2. Figure 2 depicts the docking conformations of a selected compound during the docking process. When used in conjunction with the 13 amino acid residues in the active site of the main protease, the ligand formed seven hydrogen bonds within three degrees of freedom with five amino acid residues, namely Cys 44, Asn 51, Pro 52, Tyr 54, and Arn 188 residues (Figure 2). When there are more hydrogen bonds, the binding efficiency and inhibition are both increased as a result [40]. Six-methyl-3,4-dihydro-1,2,4-triazin-5(2H)-one moiety: The sulfur atom of Cys 44 amino acid formed two hydrogen donor interactions with the nitrogen and methyl groups of the triazin-5(2H)-one moiety. During the formation of the H-donor interaction between the carbon atom of the 3-methoxycyclohex-1-ene moiety and the carbon atom of Asn 51, an H-donor interaction is formed. The nitrogen atoms of the 5-methyl-4,5-dihydro-1,2,4-triazole moiety interacted with the carbonyl oxygen of the Pro 52 and the amino group of the Arg 188 through H-donor and H-acceptor bonds formed by the amino group of the Arg 188. It has also been discovered that the nitrogen and oxygen atoms of the 6-methyl-3,4-dihydro-1,2,4-triazin-5(2H)-one moiety of the compound form H-bonds with the active residues Tyr 54 and Arg 188 of the main protease, confirming previous findings. Further, the hydrophobic interactions between His 41 and Met 49 are demonstrated (Figure 2).

**Figure 3:**
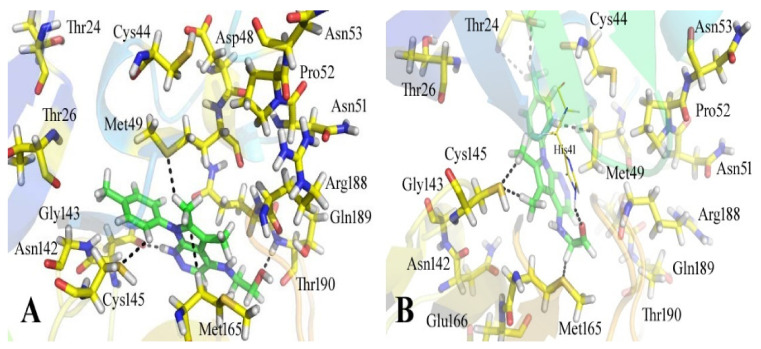
The binding style of the ZINC44928678 ZINC database hit at the M^pro^ protein’s active site (**A**) before and (**B**) after MDS.

The compound ZINC44928678 (ZINC database) had a good docking score of -41.0291 and good interactions with the target protein’s active residues. This compound formed seven polar interactions with the active site residues (Thr 24, His 41, Cys 44, Met 49, Cys 145, and Met 165) according to its binding mode (Figure 3). The carbonyl oxygen and carbon atoms of the 2-(pyridazin-4-ylamino) acetaldehyde moiety of the compound interacted with His 41 and Met 165 residues. The toluene moiety’s methyl group formed two H-donor bonds with the Thr 24 residue, while Met 49 showed H-donor interaction with the toluene moiety’s carbon atom. The dimethyl groups of the 5,6-dimethyl-7H-pyrrolo [2,3-c] pyridazine moiety of the ligand form two H-donor linkages with Cys 145.

**Figure 4:**
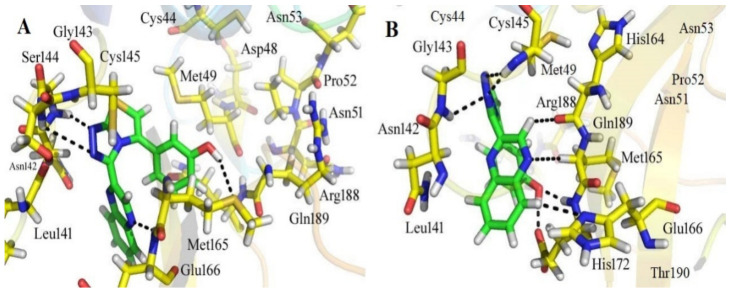
The binding mode of the 12-quinoxaline derivative of the in-house database within the active site of M^pro^ protein (**A**) before and (**B**) after MDS.

The 12-quinoxaline derivative from the in-house database had the highest activity among the compounds, with a docking score of −38.7102. It was predicted, based on the docking conformations of the ligand 12-quinoxaline derivative, that the 12-quinoxaline derivative would form hydrophilic and hydrophobic contacts with the active residues of the M^pro^ protein. These active residues include Cys 44, Met 49, Asn 51, Pro 52, Asn 53, Leu 141, Asn 142, Gly 143, Cys 145, His 164, Met 165, Glu 166, His 172, Arg 188, Gln 189, and Thr 190 of M^pro^ protein. It was revealed that the compound formed eight polar interactions with the active residues of the receptor. The H-bond was observed between -NH group of the Asn 142 and nitrogen atom of the thiazolo [2,3-c][1,2,4]triazole moiety of the inhibitor. Cys 145 residue forms two H-acceptor bonds with the nitrogen atoms of the thiazolo [2,3-c][1,2,4]triazole moiety. The carbonyl oxygen atom and –NH group of the Glu 166 residue are connected to the OH group of the phenol moiety of the hit via H-bonds. His 164, Met 165, and His 172 residues were seen to form three H-bonds with the 1,4-dihydroquinoxaline moiety of the compound, as shown in Figure 4.

**Figure 5:**
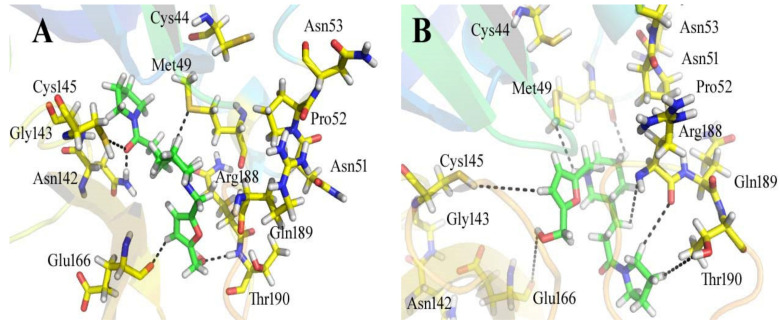
The active site of the M^pro^ protein (**A**) before and (**B**) after MDS, showing the binding mechanism of the obtained inhibitor ChemBridge63310525 from the ChemBridge database.

ChemBridge63310525 retrieved active hits from the ChemBridge database, forms seven hydrogen bonds, and has a high docking score (−38.0478). On the binding site, ChemBridge63310525 interacts with five important residues and comes out on top. Met 49, Cys145, Glu 166, Gln 189, and Thr 190 form hydrogen bonds with the moieties of the ChemBridge63310525. The hit’s furan-2-ylmethanol moiety binds to Cys 145 and Glu 166 via polar bonds. By hydrogen bonding, the 1-(pyrrolidin-1-yl)butan-1-one moiety interacts with Gln 189 and Thr 190. Met 49 forms H-bonds with the piperidin-1-ium moiety of the ligand. Asn 51, Glu166, and Pro 52 interact hydrophobically with the rest of the structure (Figure 5). The compounds that interacted more strongly than the reference inhibitor with the M^pro^ of SARSCoV-2 are listed in Table 1.

The retrieved hit compounds are powerful and polarizable due to the electronic cloud density of benzene rings (delocalized electrons), the presence of OH and NH, as well as electron repulsion groups (-nitro). All hits from the study displayed good M^pro^ interactions and docking scores. All of the lead compounds’ docking scores and 2D structures are given in Table 1.

### 3.4. MD Simulation Analysis of the Final Lead Hit/M^pro^ Complexes

**Figure 6:**
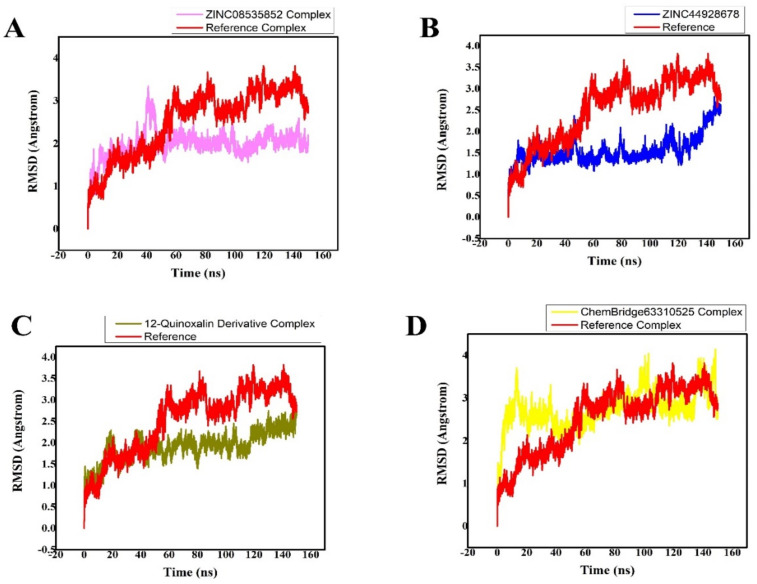
(**A**–**D**) Plot of the lead hits/M^pro^ and ref-ligand (N3)/M^pro^ complexes’ root mean square deviations.

The AMBER 18 software was used to perform a 150-ns MDS on the retrieved hit compound/M^pro^ complexes in order to determine their well-stabilized and equilibrated structures. The AMBER 18 software was used to perform MDS on the topmost active retrieved compound/M^pro^ systems, two inhibitors from the ZINC database (ZINC08535852, ZINC44928678), one hit compound from the in-house database (12-quinoxaline derivative), and one compound from the ChemBridge database (ChemBridge63310525) in complex with the M^pro^ drug target, as well as the reference-ligand (N3) M^pro^ complex. Calculating the RMSD of the backbone atoms was used to check the stability of the four finalized ligand/receptor complexes. The amplitudes of the C atoms’ fluctuation amplitudes were inversely proportional to the system’s stability. The lower the RMSD variation, the more stable the system is, and the fluctuations of the C atoms in the system [41] will be smaller. The RMSD plot is used to understand the complex’s stability, while the RMSF plot is used to understand its structural flexibility (Figure 6).

MDS equilibration was completed using the ZINC08535852/M^pro^ system at 150 ns. The RMSD graph showed that the amplitudes of the fluctuations rose with an RMSD score of 3 Å from 0 to 40 ns, and then the score decreased to 1.8 Å with slight fluctuations after 40 ns. After 140 ns up to 150 ns, the system was completely stabilized, with an RMSD of 1.8 Å and the least amount of fluctuations (Figure 6A).

The third compound is ZINC44928678 in complex with M^pro^. This complex was then subjected to the MDS to produce an energy-minimized and stabilized structure. The system’s RMSD graph revealed that the complex was stable from 0 to 100 ns of MDS at 1.5 Å, but that after MDS progression from 100 to 120 ns, their RMSD score increased to 2 Å as the system became unstable. The system was then stabilized after 120 ns, displaying smaller fluctuations with a slight increase in RMSD 2.5 Å, as shown in Figure 6B.

M^pro^ in complex with the 12-quinoxaline derivative was the second most active compound, with an RMSD of approximately 2.4 Å during the 150 ns simulation. The MDS of this ligand in contact with the M^pro^ can be shown in Figure 6C. This MDS was computed using RMSD scores of 2.4 Å, and the results are shown in Figure 6C. The RMSD graph of this system shows that the fluctuations became less and obtained an RMSD score of 2.4 Å after executing the 120–150 ns of MDS.

The ChemBridge63310525/M^pro^ (hit ligand/protein) complex’s RMSD graph displayed the highest variations from 0 to 140 ns, with an increase of approximately 3.5 Å. Soon after 140 ns of MDS, smaller fluctuations were seen in the complex system’s structural backbone, with an RMSD of approximately 2.8 Å (Figure 6D). M^pro^ undergoes conformational changes, as evidenced by the differences in the backbone RMSD in hits/M^pro^.

**Figure 7:**
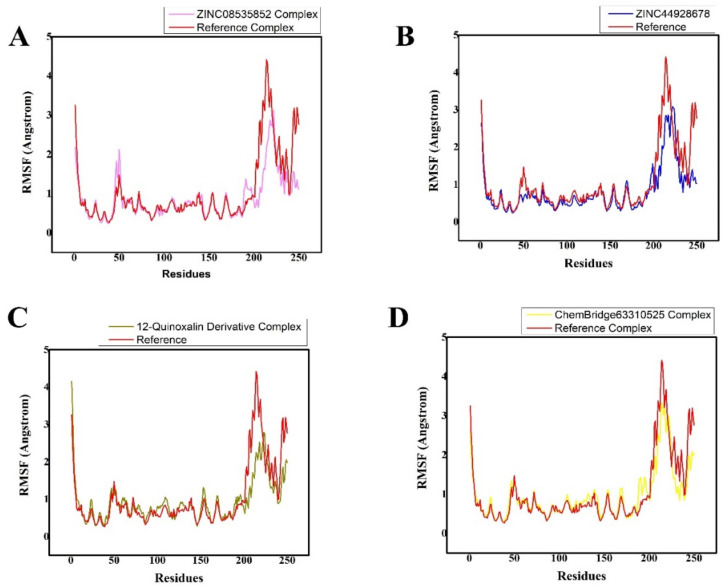
(**A**–**D**) Plot of the root mean square fluctuation of the identified lead hits/M^pro^ (purple, blue, olive green, and yellow, respectively) and ref-ligand (N3)/M^pro^ (red color) complexes. The various color schemes illustrated various hits/M^pro^ complexes and their related trajectories. Each system possessed the residual flexibility. On the graph, the number of residues is shown along the x-axis, and the RMSF is displayed along the y-axis in angstroms.

The fluctuations of the protein residues upon ligand binding, were analyzed by root mean square fluctuation (RMSF) (Figure 7A–D). The stability of a complex can be inferred from its RMSF (root mean square fluctuation) trajectories. An unstable bond is indicated by a plot with a lot of movement. A low number, on the other hand, or less fluctuation, denotes well-structured and less distorted complicated regions. RMSF is an equilibrium property that is calculated from the entire MD trajectory, so is its time average. For M^pro^ with residues containing the four possible inhibitors, the RMSF of backbone atoms was computed. The active site residues that interacted with the ligands were discovered to be stable and showed minimal variations over time, confirming the stability of the molecules with the target protein. The main variations, in contrast, were associated with regions that were far from the ligand active site, and others were discovered in the flexible loop region. Additionally, it was noted that the RMSF variations in the complexes were smaller than those in the other complex, indicating that it had substantially less structural mobility than the other complex. All of the systems exhibited patterns that were strikingly similar, as presented in Figure 7A–D. Figure 7A–D clearly show the overall RMSF value for all complexes.

### 3.5. Radius of Gyration (Rg)

**Figure 8:**
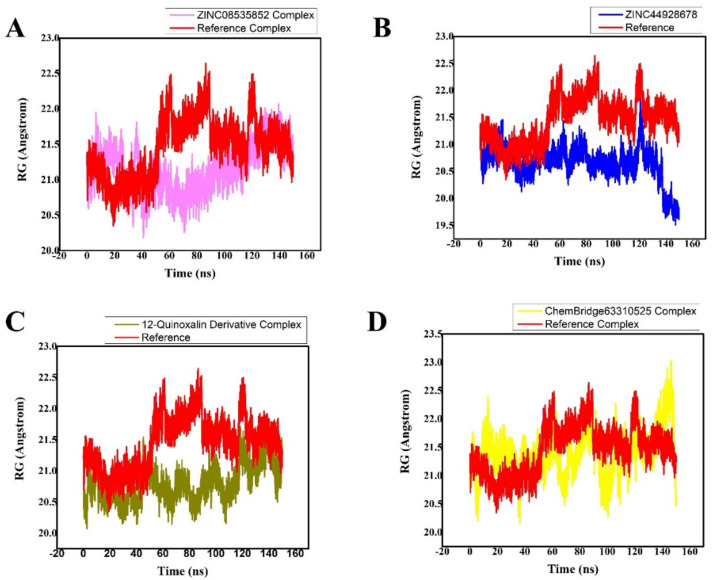
(**A**–**D**) The radii of gyration of the identified lead hits/M^pro^ (purple, blue, olive green, and yellow, respectively) and ref-ligand (N3)/M^pro^ (red color) complexes were plotted.

The relationship between Rg and time was plotted in order to determine the system’s compactness. Low Rg values are explained by the structure’s high stability and closed, compact structure, whereas high Rg values are explained by the structure’s low compactness when compared to conformational entropy (more folded). Figure 8 shows the simulation Rg values for the four compounds, which are easily discernible (as shown). In the complex, the Rg values remained stable, indicating that the binding of these molecules does not alter the protein’s structure. The retrieved four ligands/M^Pro^ complexes have shown low Rg values (compacted conformation of complexes) as compared to the reference ligand (N3)/M^Pro^, therefore, indicating that the obtained hits were active inhibitors. The Rg is a parameter for assessing the compactness and overall dimension of the protein, which in turn signifies the folding and unfolding of the protein. The lower the gyration values, the more folded the protein is, and vice versa. Therefore, Rg was calculated to determine whether the compounds/ligands maintained the folding of the system. Condensed architecture and size are supported by the complexes’ Rg range (Figure 8A–D).

### 3.6. Molecular Mechanics with Generalized Born and Surface Area Solvation (MMGBSA)

This module also looked at the binding energy calculation of selected top compounds based on their affinity for the active site binding pocket. ZINC08535852, the first lead, had an MMGBSA score of −39.5546 Kcal/mol, whereas the active lead in in-house database, 12-quinoxaline derivative, had an even lower MMGBSA score of −37.8210 Kcal/mol. The MMGBSA binding free energies of the ZINC44928678 and ChemBridge63310525 are −35.8398 Kcal/mol and −33.2041 Kcal/mol, respectively. These results lead to the conclusion that the retrieved hits had a higher free binding energy score than the reference (−20.7812 Kcal/mol) (Table 3). In order to confirm the possible uses of these powerful inhibitors in COVID-19, we will soon design further experimental studies to test the inhibitory capacity of compounds towards SARS-CoV-2.

The interaction reports of the selected hits (ZINC08535852, ZINC44928678, 12-quinoxaline derivative, ChemBridge63310525, and N3 (reference ligand)) were mentioned after the MD simulations in Table 2, while the remaining data were after the docking, i.e., the docked complexes’ interactions reports.

## 4. Conclusions

The primary goal of this study was to conduct a SBVS of compounds from the in-house, ZINC, and ChemBridge databases, as well as molecular docking and MDS of selected compounds and reference ligands (N3), as well as an estimation of binding interactions against the M^pro^ of SARS-CoV-2. Three active inhibitors, the top active one hit from each database, from the ZINC, ChemBridge, and in-house databases (ZINC08535852, ChemBridge63310525, and 12-quinoxaline derivative, respectively) showed a strong interaction against the active site of the M^pro^ of SARS-CoV-2. These compounds were identified as the most active anti-viral compounds ZINC08535852, ChemBridge63310525, and 12-quinoxaline derivative, against the M^pro^ of SARS-CoV-2. These results show that these compounds have the potential to be employed as a medication to treat SARS-CoV-2 illness. If in vitro and in vivo clinical testing are followed, the compounds could potentially serve as a candidate for the COVID-19 regimen.

## Figures and Tables

**Table 1:** The finalized lead hit compounds’ 2D structures and docking values.

S. NO	Compound Names	Structures	Docking Scores
1	ZINC08535852	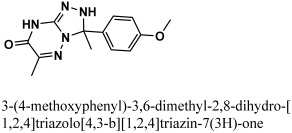	−41.3801
2	ZINC44928678	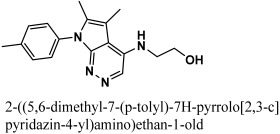	−41.0291
3	ZINC72171104	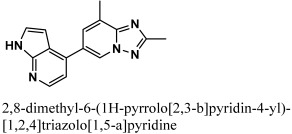	−39.5487
4	12-quinoxaline derivative	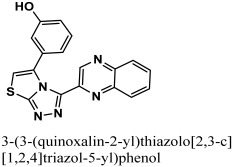	−38.7102
5	ChemBridge63310525	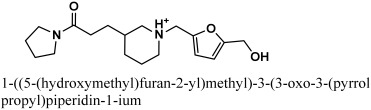	−38.0478
6	18-quinoxaline derivative	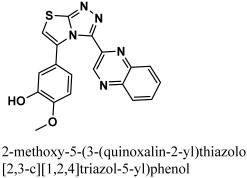	−37.5300
7	ChemBridge53208972	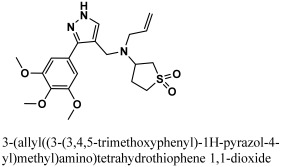	−35.4302
8	25-quinoxaline derivative	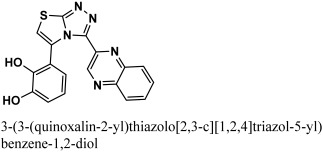	−34.3177
Reference	N3	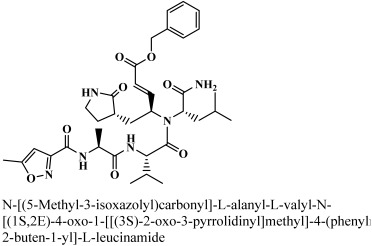	−29.5841

**Table 2:** Details of binding interactions against COVID-19 M^pro^.

Compounds IDs	Ligand	Receptor			Interaction	Distance	E (kcal/mol)
ZINC08535852	C1 1	SG	CYS	44	H-donor	3.91	−0.2
	N1 3	SG	CYS	44	H-donor	3.34	−0.6
	N4 7	O	PRO	52	H-donor	3.09	−1.1
	C6 9	O	ASN	51	H-donor	3.22	−0.1
	NH 11	O	Tyr	54	H-donor	3.69	−0.1
	N3 6	NH1	ARG	188	H-acceptor	3.01	−0.7
	O2 19	CA	ARG	188	H-acceptor	3.85	−0.1
ZINC44928678	C1 1	OG1	THR	24	H-donor	3.65	−0.1
	C1 1	O	THR	24	H-donor	3.65	−0.1
	C6 6	SD	MET	49	H-donor	3.82	−0.2
	C14 18	SD	MET	165	H-donor	3.58	−0.1
	C16 21	SG	CYS	145	H-donor	3.88	−0.1
	C17 22	SG	CYS	145	H-donor	4.1	−0.3
	O1 20	NE2	HIS	41	H-acceptor	2.98	−0.9
ZINC72171104	N2 8	SG	CYS	145	H-donor	3.33	−2.2
	N5 20	O	THR	190	H-donor	2.83	−4.7
	5-ring	CA	ASN	142	pi-H	4.1	−0.4
	5-ring	CA	MET	165	pi-H	3.51	−0.3
	6-ring	CB	MET	165	pi-H	3.59	−0.5
	6-ring	CD	PRO	168	pi-H	4.88	−0.3
	5-ring	CD	PRO	168	pi-H	4.43	−0.3
	5-ring	CA	GLN	189	pi-H	3.64	−1
	6-ring	CG	GLN	189	pi-H	4.21	−0.7
12-quinoxaline derivative	C3 3	ND1	HIE	172	H-donor	3.86	−0.2
	C24 24	O	HIE	164	H-donor	3.43	−0.3
	O25 25	OE1	GLU	166	H-donor	2.53	−4.7
	O25 25	NH	GLU	166	H-acceptor	2.4	−4.8
	N12 12	N	CYS	145	H-acceptor	3.12	−0.8
	N11 11	N	CYS	145	H-acceptor	3.12	−0.8
	N4 4	CA	MET	165	H-acceptor	3.25	−0.1
	O25 25	N	GLU	166	H-acceptor	2.86	−1.3
18-quinoxaline derivative	C3 3	SG	CYS	44	H-donor	2.97	−0.2
	O27 27	NH	ASN	142	H-donor	2.8	−0.6
	O27 27	O	GLY	143	H-acceptor	2.5	−0.9
	C24 24	NH	GLY	143	H-acceptor	1.6	−1.8
	C26 28	SG	CYS	145	H-donor	2.39	−0.1
	6-ring	CG	MET	49	pi-H	4.73	−0.1
	6-ring	CG	MET	49	pi-H	3.86	−1
	C3 3	CA	MET	49	pi-H	4.23	−0.8
25-quinoxaline derivative	C3 3	SD	MET	49	H-donor	2.84	−0.2
	O26 26	O	GLU	166	H-donor	2.7	−2.3
	O25 25	NH	GLU	166	H-donor	2.8	−2.1
	N13 13	CB	THR	190	H-acceptor	2.58	−0.2
	N7 7	CA	GLN	189	H-donor	3.36	−0.3
	N10 10	NH	ARG	188	H-acceptor	3.31	−0.1
	5-ring	N	THR	190	pi-H	3.67	−0.3
ChemBridge63310525	C9 9	O	ARG	188	H-donor	2.93	−0.5
	C11 11	NH	ARG	188	H-donor	2.5	−0.8
	C8 8	SD	CYS	145	H-donor	3.59	−0.1
	O19 19	O	GLU	166	H-donor	3.44	−0.4
	C14 14	O	MET	49	H-donor	3.13	−0.2
	C22 22	OH	THR	190	H-acceptor	2.53	−0.1
	C20 14	C	MET	49	H-donor	3.13	−0.2
ChemBridge53208972	C15 22	O	HIE	41	H-donor	3.75	−0.1
	C18 28	O	HIP	164	H-donor	3.3	−0.2
	C19 29	SD	MET	49	H-donor	4.48	−0.1
	C18 28	5-ring	HIE	41	H-pi	3.58	−0.1
	6-ring	CA	ARG	188	pi-H	4.02	−0.4
	6-ring	CD	ARG	188	pi-H	4.42	−0.1
	5-ring	CD	ARG	188	pi-H	4.52	−0.1
	6-ring	N	GLN	189	pi-H	4.76	−0.3
N3 (reference ligand)	N 13	O	THR	190	H-donor	2.85	−2.6
	N 23	O	GLU	166	H-donor	2.83	−4.8
	N 39	OE1	GLN	189	H-donor	2.93	−3.3
	O 85	N	GLY	143	H-acceptor	2.80	−1.0
	CD1 50	5-ring	HIS	41	H-pi	4.08	−0.5

**Table 3:** The average backbone RMSD and binding free energies for the best four complexes. The data are reported as the average ± standard error of the mean (SEM).

Compound Names	RMSD (Å)	Binding Free Energies (Kcal/mol)
ZINC08535852 (ZINC database)	1.8 ± 0.005	−39.5546 ± 0.3671
ZINC44928678 (ZINC database)	2.5 ± 0.005	−35.8398 ± 0.1901
12-quinoxaline derivative in-house database	2.4 ± 0.005	−37.8210 ± 0.5091
ChemBridge63310525 (ChemBridge database)	2.8 ± 0.005	−33.2041 ± 0.2102
Reference (N3)	2.8 ± 0.0126	−20.7812 ± 0.4214

## Data Availability

Data is contained within the article. The data presented in this study is available to researchers upon request.
